# Binge drinking among young adults in an urban tertiary care emergency department in Israel

**DOI:** 10.1186/s13584-017-0156-1

**Published:** 2017-07-01

**Authors:** Daphna Levinson, Paola Rosca, Doron Vilner, Idit Brimberg, Yael Stall, Ayelet Rimon

**Affiliations:** 1 Research & Planning, Mental Health Services, Ministry of Health, Jerusalem, Israel; 2 Department for the Treatment of Substance Abuse, Ministry of Health, Jerusalem, Israel & Hebrew University Jerusalem, Jerusalem, Israel; 3 Tel Aviv Sourasky Medical Center, Tel Aviv-Yafo, Israel; 4 Dana Children’s Hospital, Tel Aviv Sourasky Medical Center, Tel Aviv-Yafo, Israel; 5 Pediatric Emergency Medicine, Dana-Dwek Children’s Hospital, Tel Aviv Sourasky Medical Center, Sackler School of Medicine, Tel-Aviv University, Tel Aviv-Yafo, Israel

**Keywords:** Israel, Bing drinking, Adolescents and young adults, Emergency department, Survey

## Abstract

**Background:**

Alcohol use is a major preventable public health problem with serious health and social consequences especially among youth. In Israel, alcohol use has become an emerging problem during the last decade, and its use has increased among adolescents and young adults. Binge drinking is the common pattern of alcohol consumption among young adults who drink for recreational purposes. The present survey was conducted among 16–35 years old visitors to the ED. The aim was specifically to identify binge drinkers in order to assess the scope of the need for a brief counseling intervention among young people who arrive intoxicated to a large tertiary care urban ED in Israel.

**Methods:**

The survey was conducted throughout a 1 week period (24 h per day) at the general EDs in a large, tertiary care center, situated in Tel Aviv. During the survey week, 946 individuals, aged 16-35, visited the ED and 573 (63%) of them were approached for an interview. 89% of those approached agreed to be interviewed. Consenting patients [*N* = 348] were asked whether they drink any alcohol, how often they drink and how much.

**Results:**

About one fifth of those interviewed were in the habit of consuming more than four units of alcohol per occasion. Drinking several times a week or every day was reported by 19% of the males and 26% of the females. Frequency of the drinking episodes was highly correlated with the number of units of drink per occasion.

**Conclusions:**

The study found a very high rate of binge drinking among ED visitors, and this suggests a need for large scale ED-based interventions. As binge drinkers are at elevated risk for accidents, violence and related problems, effective ED-based interventions could make an important contribution to public health. Accordingly, Israel is in the process of assessing the effectiveness of a large-scale ED-based counseling intervention.

**Trial registration:**

Trial registration number 0230-13-TLV.

## Background

Approximately 4.5% of the global burden of disease and injury is attributable to alcohol [1]. Alcohol misuse, is also one of the leading causes of death among individuals between 12 and 20 years old [2], primarily due to unintentional injuries, homicide, and suicide [3, 4]. Thus, alcohol use is a major preventable public health problem with serious health and social consequences especially among young adults. A recent OECD report quotes data showing that admissions to hospitals for alcohol overdose have increased by 25% among 18- to 24-year-olds in the US between 1999 and 2008, [5] and a recent systematic review shows that for those born in the late 1900s there is no longer a gap between males and females in indicators of alcohol use and related harms [6].

Abusing alcohol for the purpose of getting drunk rapidly, to unwind and have fun has become the standard way of fitting in with peers. This trend is associated with an increase in morbidity and mortality from accidents, injuries, and violence and an increased exposure to the risk of many chronic conditions for young drinker [7].

In Israel, alcohol use during the last decade has increased among adolescents and young adults [8] and has become an emerging problem. The increasing number of accidents and injuries due to alcohol use among young adults and the fact that adolescents have begun drinking at a lower age, urged the Israeli Government in 2010, to launch the National Program for the Reduction of Harmful Drinking.

This initiative included amendments to the existing legislation regarding youth and alcohol [9]. Since then,

- Minors [under 18 years of age] are prohibited from consuming alcoholic drinks or possessing alcoholic beverages in an open container (bottle, can, etc.) in public places during all hours of the day.
- It is prohibited to sell, offer or serve alcoholic drinks to minors.
- It is prohibited to consume alcoholic drinks or possess alcoholic beverages in an open container in a public place between 21:00 pm and 6:00 am.
- It is prohibited to sell alcohol between the hours of 11 pm and 6 am, except in places serving alcohol for consumption in its premises (pubs, etc.).
- Police officers are granted the authority
  ○ to confiscate an open container with alcohol and pour out the contents at all hours of the day, if they feel there is a perceived threat to public safety. This applies to both adults and minors;
  ○ to close, for up to 15 days, any business that violates the prohibition to sell alcohol between 11 pm and 6 am or that sold or served alcohol to a minor.
- The Blood Alcohol Content [BAC] limit for young drivers under 24 years of age, for public transportation drivers or drivers of commercial vehicles over 3.5 tons, was lowered from 0.05 to 0.01 [9].

As part of this initiative, the Ministry of Health focused its activities on early detection and brief interventions in various health settings, with a special attention to Emergency Departments (ED) of general hospitals, where the intoxicated young first arrive.

The relatively high prevalence of alcohol use among ED patients in many western countries, the costs associated with these potentially avoidable injuries and the fact that very few episodic binge drinkers turn to consultation voluntarily, led many hospitals to initiate brief interventions [BI] within the ED’s to reduce alcohol consumption [10–17].

The rationale for these interventions is that they take place when patients see the seriousness of the event causing the admission, feel their own vulnerability and understand the role of alcohol in causing it. This was concluded from results showing that half of ED patients who drank prior to their injury attributed their injury to the alcohol and such causal attribution was associated with motivation to change alcohol use [18].

The results on the effectiveness of brief intervention for alcohol misuse in emergency departments are naturally mixed [16] given the possible different forms that the intervention can take.

Following these examples, the Ministry of Health planned to implement a similar brief counseling intervention for adolescent and young adults in a large tertiary care urban ED’s in Israel and to investigate its effectiveness.

The first question related to the effectiveness was whether there is a need for such intervention, i.e. what is the prevalence of alcohol use among visitors to the ED in Israel. It was decided to conduct a one 24/7 week survey of all young, age 16-35, visitors who arrived at the ED for any reason, prior to the initiation of the intervention project, to determine whether the alcohol drinking habits of young visitors indicate a need for such intervention.

The present paper summarizes the survey done to answer the above question. Binge drinking is defined as consuming more than four to five drinks on occasion [19] and it is the common pattern of alcohol consumption among adolescents and young adults who drink for recreational purposes [20].

Most victims of alcohol-related injuries presenting to the ED are occasional binge drinkers who are not “alcohol abusers” or “alcohol-dependent” according to DSM-IV [15], and who do not see a problem in their drinking pattern, even though it impairs cognitive functioning [21], increases the likelihood of “blackouts” (no recollection of complex activities) and puts them and others in harm’s way [20].

Previous surveys on alcohol prevalence in EDs in Israel checked the frequency of “alcohol abuse disorder” among those who arrived at the ED in a state of confusion, unconsciousness or other signs of alcohol poisoning [22–25].

The frequency of binge drinking in the general population was checked in two population surveys in Israel: Neumark et al. assessed binge drinking in 1995 on a representative sample of 18-40 years old. Among those who did not abstain from alcohol, 21% of the Arab males, 15% of the Jewish males, 7% of the Arab females and 4% of the Jewish females reported binge drinking patterns [26].

A telephone survey conducted by the Ministry of Health in 2013 on general health behaviors on a representative sample of Israeli adults aged 21 and above found that the frequency of any alcohol drinking was 58% among Jews, 53% among Christians, 15% among Druze and 4% among Moslems. Binge drinking among those drinking in the previous month was reported by 25% of the Moslems and the Druze, 13% of the Christians and 4% of the Jews [27].

The most recent HBSC survey in Israel [28] found that among the 15 years old, 15% of the boys and 5% of the girls were drunk on two or more occasions. Thus Israeli studies to date have examined the prevalence of early drunkenness and binge drinking in the general population, and the prevalence of alcohol abuse disorders among ED visitors, but not the prevalence of binge drinking among ED visitors. Accordingly, the main goal of the present survey was to assess the extent of binge drinking among young adults arriving at the ED for any reason.

## Methods

The survey was conducted at the general emergency department of the Tel Aviv Sourasky Medical Center, the biggest tertiary care center in the Tel Aviv area which is a large metropolitan area in Israel. The ED serves 140,000 adults annually.

### Sampling

All patients, 16-35 years old, admitted to the ED because of some medical problem during 1 week in May 2014 were asked to participate in the survey regardless of the reason for their admission. The specific week was chosen after inspecting the number of ED visitors ages 16-35 in the 52 weeks of 2011. The month of May 2011 had a weekly number of visitors closest to the median number. The specific week in May 2014 [11th to the 18th] was chosen because it did not have special holidays.

The sample did not include admissions that were immediately transferred to units outside the main ED such as: obstetrics, ophthalmology, ear nose and throat, oral & maxillofacial surgery and dermatology. Interviews took place while the visitor was waiting for service in the ED.

Of all the intended subjects in the main ED, only about 65% were eventually contacted to be interviewed. The other 35% were not reached by the social worker either because their ED stay was brief or because they were quickly referred to surgery.

### Consent and data collection

When presenting the study, survey staff explained that:

- the purpose of the interview was to study patterns of alcohol use among young people
- all young people attending the ED are asked to participate regardless of the reasons for their arrival
- the information collected will be used only for the survey without any disclosure of personal details, and
- that patients may refuse to answer any question or stop the interview without any consequences to the care they receive in the ED.

Consenting patients were asked whether they drink any alcohol. Those who answered positively, were further asked to describe their current habits: to quantify how much they drink when they drink, how often they drink these quantities and whether, in their opinion, the recent ED admission was related to alcohol use. The surveys were performed by hospital-based social workers, trained to present the questions in a manner that won’t intimidate the patient from complying. The study was approved by the institutional Ethics Committee.

### Analysis

Analysis consisted of frequency tables and crosstabs. The total number of units of alcohol was calculated in the following way:

$$ \mathrm{Total}\ \mathrm{number}\ \mathrm{of}\ \mathrm{alcohol}\ \mathrm{units}=1\mathrm{x}\ \left( number\  of\  small\  cans\  of\  beer\right)+1.5\mathrm{x}\ \left( number\  of\  big\  cans\  of\  beer\right)+1\mathrm{x}\ \left( number\  of\  glasses\  of\  wine\right)+1\mathrm{x}\ \left( number\ {of}^{"}{chasers}^{"}\right) $$

## Results

During the survey week, 946 individuals aged 16-35 visited the general ED. Of those, 573 (61%) were contacted for an interview.

The group that was not contacted (*n* = 373) had a similar age and gender distribution as the study group, and a slightly, but not significantly higher, percentage of all types of injuries as the presenting complaint at admission [32% vs. 28%].

Of the 573 ED visitors who were contacted for an interview, 93% were Israelis, 7% foreign workers, tourists or West Bank Palestinians. Fifty-two percent of the interviewees were males, and about half of the males and the females were younger than 27 years of age.

The presenting complaint at admission, as recorded by the registrar office, were “disease” 68%, “fall/injury” 11%, “car accident” 12%, “other accidents” (bicycle/work/burn) 5%, and “other” 4%. 11% of the group who were contacted in the ED, refused to participate in the survey. Another 2% could not participate for medical reasons.

Table 1 shows that refusal rates were higher among those who were admitted after car accidents (17%) but closer to the average (9%) among those who were admitted for all the other reasons. There were no differences in age or gender between those who agreed to participate and those who refused.

**Table 1:** Refusal to participate by initial reason for admission

Initial reason for admission	Refusals
Disease	8%
Fall/injury	9%
Car accident	17%
Accident w/bicycle	11%
All others	7%
Total	9%

About 25% of those who agreed to participate declared that they do not drink alcohol. Abstention was slightly, but significantly, higher (28%) among females (*p* = 0.054). The final sample for the main questions of the survey included 348 participants. (37% of the young people who visited the ED during the study week) after excluding 373 who were not approached, 39 who were not Israelis, 69 who refused to answer and 117 who declared that they do not drink alcohol at all.

**Fig. 1:**
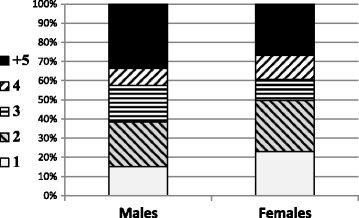
Accumulated distribution of the total number units of drink per occasion by gender

Figure 1 presents the accumulated distribution of the total number units of drink per occasion by gender. Figure 1 shows that 39% of the males and 50% of the females drink up to two units of alcohol per occasion, and a total of 67% of the males and 73% of the females drink up to four units per occasion. Binge drinking, i.e. drinking more than four units during one occasion, was reported by 35% of the males, and by 25% of the females.

**Fig. 2:**
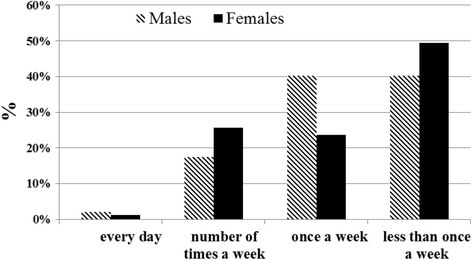
Frequency of drinking occasions by gender

The majority of participants admitted to drinking alcohol either once a week or less (Fig. 2). 19% of the males and 26% of the females responded that they drink several times a week or every day.

**Fig. 3:**
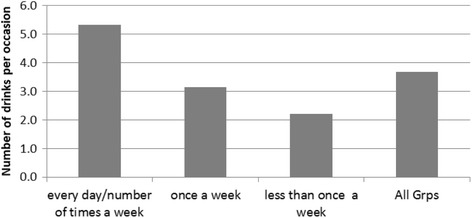
Average number of drinks per occasion for frequent and less frequent drinkers

The frequency of the drinking episodes was highly correlated with the number of drinks per occasion (Fig. 3). Those who drink a few times a week consume an average of five drinks per occasion whereas those who drink less than once a week drink on average only two drinks per occasion. The correlation was highly significant (F = 25.9, df = 2, *p* = 0.000).

## Discussion

This study was the first to estimate the extent of binge drinking among the 16-35 age group admitted to an urban ED in Israel. The findings show that this age group can be divided into 25% who abstain from drinking, 53% who were low-risk drinkers and 22% who are in the habit of consuming more than four units of alcohol per occasion.

Based on the number of alcohol units only, our estimates are close to those found in two other studies from the US which used self-report data by the general population of visitors to ED departments.

Blow et al. [29] approached all 19-60 years old who entered the ED to complete a self-report screening instrument before recruiting them to a study. The screener included a question about binge drinking in the previous 30 days and 22% of the 14,557 participants reported such behavior in the previous month.

Sommers et al. [30] approached all 18-44 years old who entered the ED to complete a self- report screening instrument before recruiting them to a study on dangerous driving. The screener defined hazardous drinking as either (i) ≥ 4 standard alcoholic drinks on a typical drinking day; or (ii) ≥ 5 drinks/occasion for women and ≥6 for men; or (iii) ≥ 11 drinks/wk. for women and ≥14 for men. 21.7% of the 8098 participants screened positive for binge drinking.

The results of the survey also show that those who binge, tend to do so more frequently than once a week. These results replicate results from the US where it was demonstrated that those who drink more than eight drinks per occasion were also more likely to be those who had more drinking occasions in the previous 30 days [31].

There are several limitations to our study. First, our results are based on self-report about habitual drinking behavior. Subjects may have denied any drinking if admitting such behavior constitutes a problem for them, or they may have overstated the number of drinks if such behavior seems admirable to them. However, we know that self-report on the number of alcohol drinks prior to an injury was verified many times using biological specimens obtained at the time of the ED referral and was found to be an accurate indicator of alcohol use [32, 33]. We therefore assume that our question on general drinking habits can provide a relatively valid measure of the subjects’ drinking habits.

Yet, since the reports on the number of alcohol drinks were based on number of “glasses” or cans without specifying size of glass or percent of alcohol content in the specific drink, the actual amount of alcohol consumed could be only roughly correlated with the number of alcohol drinks.

The second issue is the representativeness of the final sample of the study. We know that only about 65% of the intended subjects were approached to be interviewed. The other 35% were not reached by the social worker either because their ED stay was brief or because they were quickly referred to surgery. We know that the age or gender distribution of the patient group that wasn’t reached was no different from that of the final sample.

We also know that among those who were approached by the interviewer, about 10% refused to be interviewed when they heard that the subject of the survey was alcohol use, and noted that the refusal rate was higher among those who were admitted after car accidents. One possible explanation for the refusal could be that for those who are still overwhelmed from an accident or another injury, it is easier to refuse participation rather than to engage in any conversation. Another possible cause could be that if alcohol use is detected after involvement in a car accident, the insurance will not cover any expense. Hence, it is possible that alcohol was the underlying cause for the admission to the ED and consequently for the refusal to participate in the survey.

The higher proportion of injuries in the group that was not reached and the higher proportion of accidents among those who refused to answer lead us to conclude that the present results may underestimate the size of the group who binge drink.

The dangers of binge drinking were detailed earlier and include “blackouts”, low mood states, impulsive behaviors and impaired cognitive functioning. Young males and females who binge drink are also more likely to attempt suicide, suffer from depression or other mental disorder, engage in unsafe sex and are more likely to be victimized [34]. The implications of this high consumption are even more serious for females. The same amount of alcohol ingested will result in a higher concentration of alcohol in women’s blood [35], a more rapid pace towards addiction and may increase the dangers of being a victim to violence or sexual violence.

The high rates of alcohol consumption and binge drinking found in this study represent the Tel Aviv area which attracts those who want to party and drink. It is quite likely that in other cities in Israel there will be fewer binge drinkers entering the ED’s. These ED’s could also benefit from intervention designed to make drinkers more careful, and it would be useful to conduct similar studies in other cities to assess the local prevalence of binge drinking among ED visitors.

The most important implication of this survey, is that some form of ED intervention when possible is urgent, not only because of the more frequent potential for accidents and violence, but also because of the emotional problems that are more prevalent among binge drinkers.

Reports on the efficacy of BI’s on ED patients with alcohol misuse are mixed [16]. Brief interventions were shown to be beneficial for subgroups such as those who report driving accidents [36], or those with alcohol related injuries [37]. Another possible reason for the mixed results is the exclusive focus on alcohol use, without examining the possible use of other drugs prior to the admission to the ED [29].

In summary, the results emphasize the importance of directly assessing adolescents and young adults regarding alcohol use, even when visiting the ED due to complaints that seem unrelated to risky behavior. Identifying these cases is a key step to implementation of screening and brief alcohol intervention in routine practice.

## Conclusions

The high rate of binge drinking among young ED visitors, the higher risk of accidents and injuries associated with this behavior and the possibility that binge drinking indicate underlying emotional problems, stress the importance of having a brief ED intervention while the alcohol effects are still visible.

## Abbreviations

*ED*: emergency departments

## References

[1] Global status report on alcohol and health. WHO; 2011. www.who.int/substance_abuse/publications/global_alcohol_report/msbgsruprofiles.pdf.

[2] Zerhouni O, Bègue L, Brousse G, Carpentier F, Dematteis M, Pennel L, Swendsen J, Cherpitel C (2013). Alcohol and violence in the emergency room: a review and perspectives from psychological and social sciences. Int J Environ res Public Health.

[3] Graham K, Rehn N, Sempos CT, Jernigan D, Rehm J, Room R, Monteiro M, Gmel G (2003). Alcohol as a risk factor for global burden of disease. Eur Addict res.

[4] Rehm J, Taylor B, Patra J (2006). Volume of alcohol consumption, patterns of drinking and burden of disease in the European region 2002. Addiction.

[5] OECD/WHO (2015). Promoting health*,* preventing disease*:* the economic case.

[6] Slade T, Chapman C, Swift W, Keyes K, Tonks Z, Teesson M (2016). Birth cohort trends in the global epidemiology of alcohol use and alcohol-related harms in men and women: systematic review and metaregression. BMJ Open.

[7] Sassi F (2015). Tackling harmful alcohol use: economics and public health policy.

[8] Alcohol use among children and youth. The Knesset center for research and information 2010. [Hebrew]. https://www.knesset.gov.il/mmm/data/pdf/m02406.pdf. Accessed 6 June 2017.

[9] Alcohol Legislation http://www.antidrugs.org.il/english/template/default.aspx?catid=406. Accessed 6 June 2017.

[10] Cherpitel CJ, Ye Y, Bond J, Rehm J, Poznyak V, Macdonald S, Stafström M, Hao W (2005). Multi-level analysis of alcohol-related injury among emergency department patients: a cross-national study. Emergency room collaborative alcohol analysis project (ERCAAP) and the WHO collaborative study on alcohol and injuries. Addiction.

[11] Gmel G, Bissery A, Gammeter R, Givel JC, Calmes JM, Yersin B, Daeppen JB (2006). Alcohol-attributable injuries in admissions to a swiss emergency room--an analysis of the link between volume of drinking, drinking patterns, and preattendance drinking. Alcohol Clin exp res.

[12] Taggart IH, Ranney ML, Howland J, Mello MJ (2013). A systematic review of emergency department interventions for college drinkers. J Emerg Med.

[13] Coulton S, Perryman K, Bland M, Cassidy P, Crawford M, Deluca P, Drummond C, Gilvarry E, Godfrey C, Heather N, Kaner E, Myles J, Newbury-Birch D, Oyefeso A, Parrott S, Phillips T, Shenker D, Shepherd J (2009). Screening and brief interventions for hazardous alcohol use in accident and emergency departments: a randomised controlled trial protocol. BMC Health Serv res.

[14] Chun TH, Spirito A, Rakowski W, D'Onofrio G, Woolard RH (2011). Beliefs and practices of pediatric emergency physicians and nurses regarding counseling alcohol-using adolescents: can counseling practice be predicted?. Pediatr Emerg Care.

[15] Mabood N, Ali S, Dong KA, Wild TC, Newton AS (2013). Experiences of pediatric emergency physicians in providing alcohol-related care to adolescents in the emergency department. Pediatr Emerg Care.

[16] Wojnar M, Jakubczyk A (2014). Brief interventions for hazardous and harmful alcohol consumption in accident and emergency departments. Front Psychiatry.

[17] Madras BK, Compton WM, Avula D, Stegbauer T, Stein JB, Clark HW (2009). Screening, brief interventions, referral to treatment (SBIRT) for illicit drug and alcohol use at multiple healthcare sites: comparison at intake and 6 months later. Drug Alcohol Depend.

[18] Barnett NP, Apodaca TR, Magill M, Colby SM, et.al. Moderators and mediators of two brief interventions for alcohol in the emergency department. Addiction 2010 Mar;105(3):452-465. 10.1111/j.1360-0443.2009.02814.x PMC2858352 20402989

[19] Alcohol Alert. U.S. Department of Health & Human Services National Institutes of Health National Institute on Alcohol Abuse and Alcoholism, No.65 April 2005.http://pubs.niaaa.nih.gov/publications/aa65/AA65.pdf. Accessed 6 June 2017.

[20] Miller JW, Naimi TS, Brewer RD, Jones SE (2007). Binge drinking and associated health risk behaviors among high school students. Pediatrics.

[21] Courtney KE, Polich J (2009). Binge drinking in young adults: data, definitions, and determinants. Psychol Bull.

[22] Neumark YD, Yuffe-Sheinman H, Glasser J, Bar-Hamburger R, Tulchinsky T (2002). Drug and alcohol-related emergency department visits: results of a pilot survey in two hospitals in Israel. Isr J Psychiatry Relat Sci.

[23] Kozer E, Bar-Hamburger R, Rosenfeld NY, Zdanovitch I, Bulkowstein M, Berkovitch M (2008). Illicit drug and alcohol users admitted to the pediatric emergency department. Isr med Assoc J.

[24] Kozer E, Bar-Hamburger R, Rosenfeld N, Dalal I, Landu O, Fainmesser P, Ben-Yehuda Y, Berkovitch M (2009). Strategy for increasing detection rates of drug and alcohol abuse in paediatric emergency departments. Acta Paediatr.

[25] Shavit I, Konopnicki M, Winkler K, Neuman G, Kozer E, Bentur Y (2012). Serum glucose and electrolyte levels in alcohol-intoxicated adolescents on admission to the emergency department: an unmatched case-control study. Eur J Pediatr.

[26] Neumark YD, Rahav G, Jaffe DH (2003). Socio-economic status and binge drinking in Israel. Drug Alcohol Depend.

[27] Survey of Knowledge, Attitudes, and Health Practices. ICDC – Israel Center for Disease Control, Ministry of Health: [Hebrew, in print]; 2013.

[28] Growing up unequal: gender and socioeconomic differences in young people's health and well-being. Health Behaviour in School-aged Children (HBSC) study: international report from the 2013/2014 survey http://www.euro.who.int/en/publications/abstracts/growing-up-unequal.-hbsc-2016-study-20132014-survey. Accessed 6 June 2017.

[29] Blow FC, Walton MA, Barry KL, Murray RL, Cunningham RM, Massey LS, Chermack ST, Booth BM (2011). Alcohol and drug use among patients presenting to an inner-city emergency department: a latent class analysis. Addict Behav.

[30] Sommers MS, Lyons MS, Fargo JD, Sommers BD, McDonald CC, Shope JT, Fleming MF (2013). Emergency department-based brief intervention to reduce risky driving and hazardous/harmful drinking in young adults: a randomized controlled trial. Alcohol Clin exp res.

[31] Naimi TS, Nelson DE, Brewer RD (2010). The intensity of binge alcohol consumption among U.S. adults. Am J Prev med.

[32] Treno AJ, Gruenewald PJ, Johnson FW (1998). Sample selection bias in the emergency room: an examination of the role of alcohol in injury. Addiction.

[33] Cherpitel CJ, Borges G (2002). Substance use among emergency room patients: an exploratory analysis by ethnicity and acculturation. Am J Drug Alcohol Abuse.

[34] Stolle M, Sack PM, Thomasius R (2009). Binge drinking in childhood and adolescence: epidemiology, consequences, and interventions. Dtsch Arztebl Int.

[35] Cook LJ (2004). Educating women about the hidden dangers of alcohol. J Psychosoc Nurs Ment Health Serv.

[36] Mello MJ, Nirenberg TD, Longabaugh R (2005). Emergency department brief motivational interventions for alcohol with motor vehicle crash patients. Ann Emerg med.

[37] Field C, Caetano R (2010). The effectiveness of brief intervention among injured patients with alcohol dependence: who benefits from brief interventions?. Drug Alcohol Depend.

